# 

**Published:** 1852-08

**Authors:** 


### CONTENTS.

ORIGINAL COMMUNICATIONS:— PAGE.

ART. 1.—Hydrothorax, by Ira Manley, M.D., - - - - - 145
ART. 2.—Successful Removal of a Foreign Body from the Knee joint—Anomalous Character of the Same, by J. Washington Smith, - - 147
ART. 3.—Observations on some Peculiar Varieties of "After Pains," by C. Joel Hendrick, of Auburn, Ind., - - - - - - - 151
ART. 4.—Case of Uterine Hæmorrhage, consequent upon want of Contractile Power in the Uterus, terminating fatally, by A. M. Dunton, M.D., of Plover, Wis., - - - - - - - - - 152
ART. 5.—Case of Strangulated Hernia, not attended by the usual symptoms, by Samuel W. Ritchey, of Newtown, Ind. - - - - - - 154

SELECTIONS:—

On the Treatment of Cancer by the Lactate of Iron, taken by the mouth and injected into the veins, - - - - - - - - - - 157
Case of the Resection of the Superior Maxillary and Malar Bones, - - 160
Democracy and Doctors, - - - - - - - - - - 161
On Bronchitis, - - - - - - - - - - - - 165
On the Bite of a Rattlesnake, - - - - - - - - - - 168
On the Use of Glycerine in the Treatment of Certain Forms of Deafness, 171
On Cod-Liver Oil in Phthisis, - - - - - - - - - 172
Death from Hæmorrhage Consequent upon Lancing the Gums, - - 174
On Flexion of the Limbs as a Means of Suspending and even Arresting Arterial Hæmorrhage, - - - - - - - - - - 175
Death from a Dose of Urine, - - - - - - - - - 176

EDITORIAL:—

"Contributions to Experimental Physiology," by Bennet Dowler, M.D., Corresponding Member of the Academy of Natural Sciences of Philadelphia, Fellow of the Medico-Chirurgical College of the same city, &c., 177
Amputation of Lower Jaw, with Disarticulation of both Condyles—On the Claims of Priority in the Exsection and Disarticulation of the Lower Jaw—An Address to the Graduates of the Medical Department of the St. Louis University—Practical Chemistry a Branch of Medical Education—An Essay on Empirical Remedies, - - - - - 182
The Iowa University again, - - - - - - - - - 184
The Third Annual Announcement of the College of Medicine and Surgery of the University of Michigan, - - - - - - - - - 187
Whiteside County Medical Society, - - - - - - - 191
Medical News, - - - - - - - - - - - - - 192

